# Root System Scale Models Significantly Overestimate Root Water Uptake at Drying Soil Conditions

**DOI:** 10.3389/fpls.2022.798741

**Published:** 2022-02-14

**Authors:** Deepanshu Khare, Tobias Selzner, Daniel Leitner, Jan Vanderborght, Harry Vereecken, Andrea Schnepf

**Affiliations:** ^1^Institute of Bio-Geosciences (IBG-3, Agrosphere), Forschungszentrum Jülich GmbH, Jülich, Germany; ^2^Simulationswerkstatt, Leonding, Austria

**Keywords:** benchmark C1.2, hydraulic conductivity drop, multi-scale model, root water uptake, impact of grid size, Functional-structural root architecture models, Grid convergence study

## Abstract

Soil hydraulic conductivity (*k*_*soil*_) drops significantly in dry soils, resulting in steep soil water potential gradients (*ψ*_*s*_) near plant roots during water uptake. Coarse soil grid resolutions in root system scale (RSS) models of root water uptake (RWU) generally do not spatially resolve this gradient in drying soils which can lead to a large overestimation of RWU. To quantify this, we consider a benchmark scenario of RWU from drying soil for which a numerical reference solution is available. We analyze this problem using a finite volume scheme and investigate the impact of grid size on the RSS model results. At dry conditions, the cumulative RWU was overestimated by up to 300% for the coarsest soil grid of 4.0 cm and by 30% for the finest soil grid of 0.2 cm, while the computational demand increased from 19 s to 21 h. As an accurate and computationally efficient alternative to the RSS model, we implemented a continuum multi-scale model where we keep a coarse grid resolution for the bulk soil, but in addition, we solve a 1-dimensional radially symmetric soil model at rhizosphere scale around individual root segments. The models at the two scales are coupled in a mass-conservative way. The multi-scale model compares best to the reference solution (−20%) at much lower computational costs of 4 min. Our results demonstrate the need to shift to improved RWU models when simulating dry soil conditions and highlight that results for dry conditions obtained with RSS models of RWU should be interpreted with caution.

## 1. Introduction

Most functional-structural root architecture models used to calculate root water uptake (RWU) consider root system architectures (RSA's) as networks of discrete cylindrical tubes embedded in 3D soil domains. We refer to those macroscopic models as models on the root system scale (RSS) (Schroeder et al., 2009a). Approaching RWU as 1D-3D mixed-dimension coupled problem is computationally more efficient than explicitly considering the physical presence of roots and their respective volumes (Koch et al., 2018). RWU is calculated based on the water potential (*ψ*) difference between soil and xylem (Dunbabin et al., 2013). When only below-ground organs are explicitly modeled, the water potential at the root-soil interface *ψ*_*RSI*_ is defined by transpirational demand prescribed at the root collar, soil water status, and soil and root hydraulic properties. If the soil becomes dry due to RWU, k_soil_ becomes very low, leading to the formation of steep microscopic gradients in Ψ_s_ around the roots. These gradients are often not spatially resolved by the numerical grid used to simulate the soil water flow (Schroeder et al., 2008; Carminati et al., 2020; Rodriguez-Dominguez and Brodribb, 2020). The simulated *ψ*_*RSI*_, which represents the *ψ*_*s*_ that is “felt” by plants and determines their water status, is influenced by the precision with which these gradients are modeled, as *ψ*_*RSI*_ is heavily dependent on *k*_*soil*_. When accurately captured, the *k*_*soil*_ drop leads to an earlier onset of drought stress, while inaccurate representation may lead to the overestimation of simulated RWU.

Our preliminary solution of an RWU scenario in dry loam for the collaborative benchmark initiative of functional-structural root architecture models launched by Schnepf et al. (2020) prompts this study. Although we were aware of the grid size dependency of our RSS model under dry conditions (Schroeder et al., 2009b), we wanted to test it in detail for this benchmark. We found a rather large overestimation in our RWU calculations compared to the numerical reference solution computed on a fine adaptive soil grid meshed around an explicitly modeled 3D RSA. The resolved interface method used to create the reference is described in Koch (2021). Here, we develop the perspective that for drying soils, RSS modeling approaches are not suitable to capture the drop in *k*_*soil*_ satisfactorily and are, therefore, prone to numerical errors. Grid refinement may be used to increase accuracy: in dry soils, the steep part of *ψ*_*s*_ gradients extends only to a few millimeters around the roots (Schroeder et al., 2008; Metselaar and De Jong van Lier, 2011; Carminati et al., 2016), and thus, soil grid sizes similar to the root diameters are needed to resolve them (Koeppl et al., 2018; Koch et al., 2020b). However, when the soil grid resolution approaches the diameter of the roots, the physical presence of root segments can no longer be neglected (Mai et al., 2019). While methods exist to distribute the sink term across several soil elements when the grid size becomes smaller than the root diameter (Koch et al., 2021), the problem of high problem computational cost remains. To quantify the impact of grid size on RWU from dry soil, we simulate benchmark C1.2 (Schnepf et al., 2020) using our RSS model with different grids and compare the results to the reference solution. Furthermore, we implement an alternative approach by Mai et al. (2019) to show that specialized models developed to represent gradients in *ψ* within the soil element are required for a correction of *ψ*_*RSI*_ in a practice-relevant manner. This continuum multi-scale model represents water fluxes and potentials in the rhizosphere by a 1D radially symmetric model in which fluxes and potential gradients in the axial direction are neglected. This may be justified for small root segments when the gravitational head differences on the length scale of the root segment are small compared to the radial gradients (Roose and Fowler, 2004; Schroeder et al., 2009b; Mai et al., 2019), and we use this simplification to be able to reduce the problem to a 1D radially symmetric one. Note that gravity is only neglected in the 1D radially symmetric models. On the RSS, gravity is considered. Ultimately, the multi-scale model allows finer soil resolutions in the radial direction of root segments while keeping the computational costs low. We then use the reference solution to evaluate the results of the RSS and multi-scale model for loam. Finally, we extend the benchmark scenario to investigate the impact of grid size on the *k*_*soil*_ drop for the soil textures clay and sand.

## 2. Materials and Methods

### 2.1. Benchmark Scenario C1.2

The benchmark scenario is implemented based on section 2.5.5 of Schnepf et al. (2020) and considers the RWU of an 8-day-old lupine with static RSA (Supplementary Figure S1) and constant root hydraulic properties. Axial conductivities are set to 4.32x10^−2^ cm^3^/day, and radial conductivities are set to 1.72x10^−4^ 1/day. Root segment diameters range from 0.02 to 0.32 cm, with an average diameter of 0.13 cm; total root length is 53.08 cm; mean root surface area is 21.68 cm^2^. A sinusoidally modulated potential transpiration (*T*_*pot*_) rate of 6.4 cm^3^/day derived from experimental data is prescribed over a simulation period of 3 days. The 3D soil domain surrounding the RSA has dimensions of 8 x 8 x 15 cm^3^ and is parameterized as loamy soil. Used soil hydraulic properties are given in Supplementary Table S1. Assuming a hydrostatic equilibrium, the initial *ψ*_*s*_ is set at *ψ*_*s,top*_ = −659.8 cm at the soil surface.

### 2.2. Model Description

The soil water flow equations are solved using an open-source simulation framework, DuMu^*X*^ (Koch et al., 2020a), available through a python binding within our dedicated root-soil inter-actions module DuMu^*X*^-ROSI. The 3D soil domain is discretized using structured grids consisting of equally sized cuboids in which the root axes network, which is represented by a discrete network of linear 1D segments, is embedded. In the RSS model, the physical presence of roots in soil is neglected. Following, we will refer to root axis segments as root segments where each root segment has next to its spatial coordinates, root radius, and radial and axial conductivities as attributes. Soil water flow is described by the Richards equation (Richards, 1931), and the hybrid analytical solution of Meunier et al. (2017) is used for solving the water flow in the roots. RWU is calculated based on the potential difference between root-soil interface and the xylem. We solve the governing partial differential equations using a fully implicit time integration scheme coupled with the finite volume method using a cell-centered two-point flux approximation which holds the mass conservation in each control volume of soil and root. In the RSS model, *ψ*_*RSI*_ is approximated by the mean *ψ*_*s*_ of the voxels in which the root segment is embedded. In contrast, the continuum multi-scale approach of Mai et al. (2019) solves the 3D Richards equation on the RSS scale, coupled with a 1D radially symmetric model of soil water flow (1D Richards equation) that is applied on the single-root scale for each root segment. To set up the 1D single-root models, the soil voxel volume, *V*_*s*_, is divided between all root segments within the voxel proportional to their volume, *V*_*rs,i*_, and the total root volume inside the voxel, *V*_*rst*_. The soil volume assigned to a segment is calculated by $\frac{V_{rs,i}}{V_{rst}}*V_{s}$, which is then used to define its surrounding hollow soil cylinder as $\left(r_{1}^{2}-r_{0}^{2}\right)\pi L$, where the segment radius *r*_0_ and the soil cylinder *r*_1_ are the inner and outer boundaries of the 1D radially symmetric single-root model, and *L* is the segment length. At the inner boundary, water flux is prescribed based on the gradient between *ψ*_*xylem*_ and *ψ*_*RSI*_. The net flux into or out of the soil voxel on the RSS is partitioned between the root segments inside this voxel in proportion to their surface area and prescribed as flux boundary condition at the outer boundary. Distributing the RSS net flux between all soil cylinders in a soil voxel couples both models in a mass conservative way. More details on the multi-scale approach are given in Mai et al. (2019). Both modeling approaches use no-flux boundary conditions at the top and bottom boundaries of the soil domain. We assume zero flux boundary conditions at the root tips and prescribe *T*_*pot*_ as flux boundary condition at the root collar. As the *ψ*_*collar*_ reaches a threshold (−15290 cm), the boundary conditions are switched and the collar potential is set at this threshold. Equations of water flow in soil and roots are given in Schnepf et al. (2020). All simulations were performed on a local machine with an Intel® Core™ i5-8365U CPU (@1.6 GHz, 8 Cores) and 16 GB of RAM.

### 2.3. Impact of Grid Size

The RSS model was simulated at uniform soil resolutions ranging from a coarse grid of ≈ 4.0 cm to a comparatively fine grid of ≈ 0.2 cm with soil grids consisting of equally sized cuboids with almost same edge-lengths in XYZ directions. We idealize them to be cubic and give one approximated edge-length to denote the grid size (Supplementary Table S2). The 1D cylinders of the multi-scale model are discretized by 60 elements each while using a logarithmic scaling with grading factor of 1.5 (40,000 elements in total). Hence, we achieve the highest spatial resolution close to the root surface. The mean edge length is 0.08 mm, the minimum is 6.8x10^−3^ mm, and the maximum is 3.2 mm. The adaptive grid of the reference is gradually refined toward the roots and consists of 1.45 million tetrahedral cells with a mean edge length of 1.06 mm, a minimum of 3.46x10^−4^ mm, and a maximum of 3.16 mm. A comparison of 1D and reference grid is given in Supplementary Figure S2. RWU is simulated with RSS and multi-scale model and results are compared to the numerical reference solution. Relative error (*RE*), defined as $\frac{\left(f_{i}-f\right)}{f}$, is used to quantify the differences to the reference where *f*_*i*_ is the cumulative transpiration (*T*_*cum*_) at the “i^*th*^” soil resolution, and *f* is the *T*_*cum*_ of the reference solution.

Based on root hydraulic architecture, we compute the standard uptake fraction (SUF) of each root segment, yielding the relative contribution of each root segment to RWU in case of a uniform *ψ*_*s*_. These SUF values are used as weighing factor to calculate the weighted average of *ψ*_*s*_ of voxels containing root segments (Couvreur et al., 2012; Meunier et al., 2017). The resultant weighted average, known as equivalent soil water potential, *ψ*_*s,eq*_, is a metric that represents the actual *ψ*_*s*_ “sensed” by the plant. We also calculate *ψ*_*s,bulk*_ as the average of all soil elements within the domain.

## 3. Results

**Figure 1:**
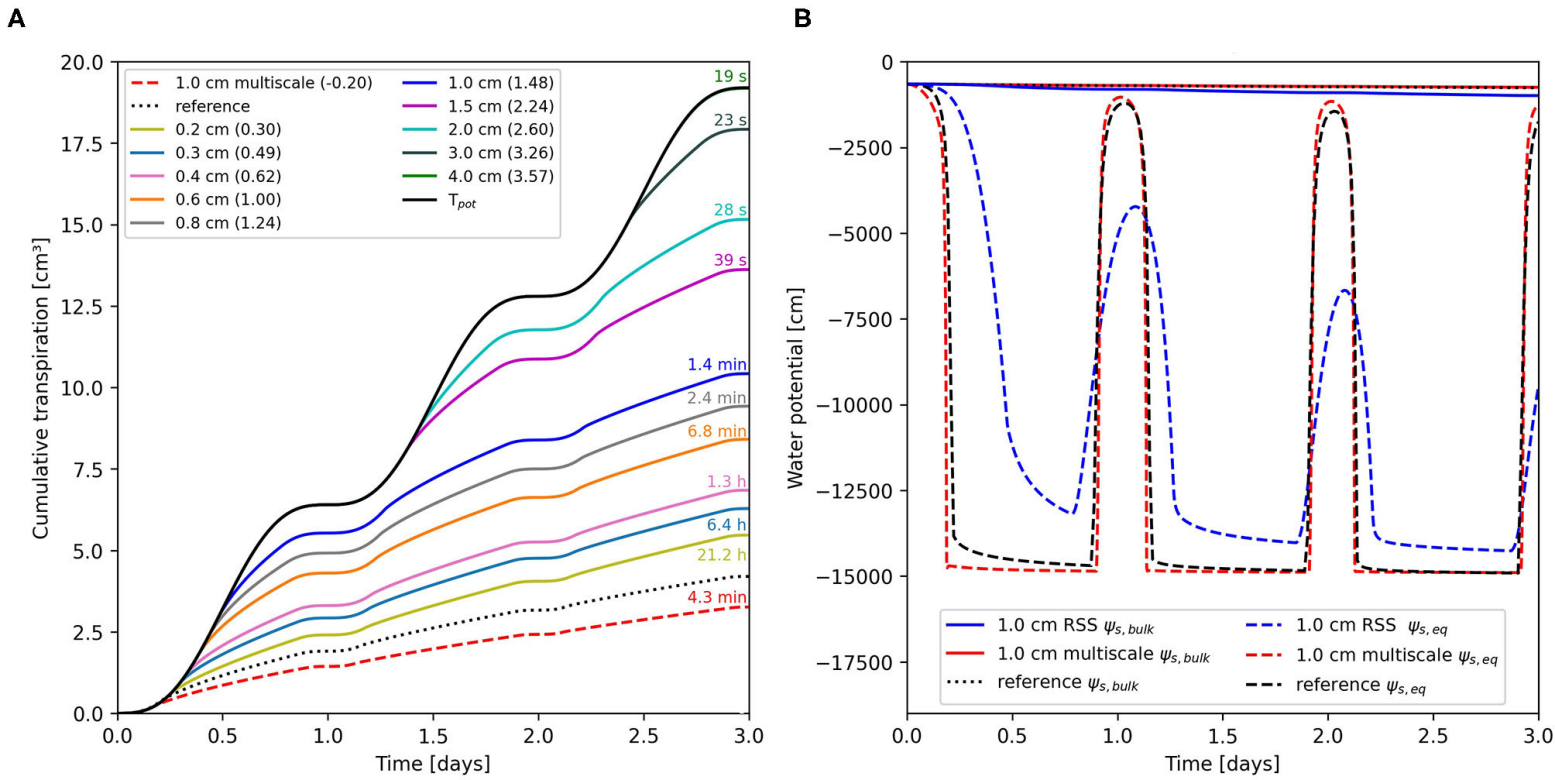
**(A)** Cumulative transpiration of the numerical reference solution (dotted), the multi-scale model (dashed), and the root system scale (RSS) model at different soil resolutions (solid) for the loamy soil scenario benchmark C1.2 at initial *ψ*_*s,top*_ = −659.8 cm. Values in parentheses indicate the relative error (RE), numbers above lines indicate the wall-clock time required to compute the respective simulation. **(B)** Equivalent soil water potential, *ψ*_*s,eq*_, (dashed) for the reference solution (black), and for RSS (blue), and multi-scale model (red) while using a bulk soil resolution of 1 cm. Blue and red solid lines show the mean bulk soil water potential, *ψ*_*s,bulk*_, of the soil domain in RSS and multi-scale model, *ψ*_*s,bulk*_ of the reference solution is shown with a black dotted line.

Results of the RSS model at different soil grid resolutions and the multi-scale model for benchmark C1.2 are shown in Figure 1A. The solid black line shows the potential *T*_*cum*_ resulting from a mean *T*_*pot*_ rate of 6.4 cm^3^/day per plant. For the given RSA with a root surface area of 24.42 cm^2^, this is equivalent to a mean water flux at the root surface of 3.42x10^−6^ cm/s which is a typical value, refer to e.g., Roose et al. (2001); Nye and Marriott (1969). For the RSS model, no stress is observed for a 4.0 cm grid. Starting at a soil resolution of 3.0 cm, drought stress is observed and with further refinement, its onset is shifted to earlier times decrease from 3.26 at 3.0 to 0.30 at 0.2 cm. The needed wall-clock times range from 19 s to 21.2 h. Applying the multi-scale model results in a *T*_*cum*_ of 3.2 cm^3^ after 3 days. In comparison to the numerical reference solution, we observe a RE of −0.20 with a required computation time of 4.3 min. As the reference solution was computed externally on different hardware, we cannot give a comparable wall-clock time.

While an initial *ψ*_*s,top*_ of −659.8 cm in a loamy soil might not seem very dry from an experimental standpoint, our simulations show that considering rhizosphere gradients will lead to uptake limitations rather quickly. A visualization of the simulated *ψ*_*s,bulk*_ and the *ψ*_*s,eq*_ sensed by the roots for reference, RSS and multi-scale model is given in Figure 1B. Although we apply a 1 cm grid on the RSS in both approaches, the gradients in the soil differ substantially. Quicker water replenishment in the vicinity of the roots leads to smaller *ψ* gradients between RSI and bulk soil in the RSS model. Utilizing the multi-scale approach results in sharper *k*_*soil*_ drops that are formed faster and result in larger *ψ*_*s*_ gradients. An additional plot of *ψ*_*s,eq*_ showcasing the transition from non-stressed to stressed conditions for multi-scale and RSS model is shown in Supplementary Figure S3.

**Figure 2:**
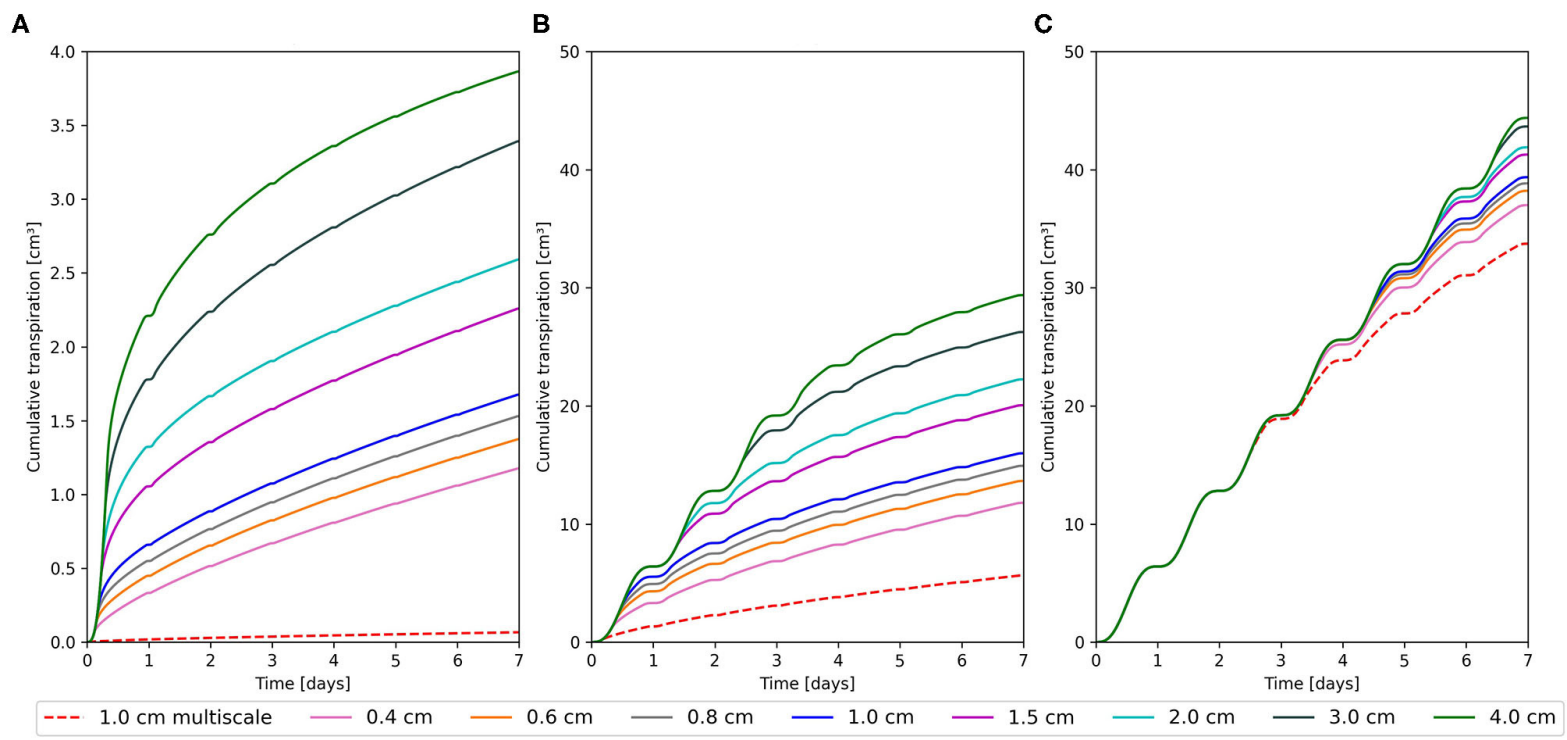
Cumulative transpiration at different soil grid resolutions for the root system scale (RSS) model (solid) and the multi-scale model (dashed). **(A)** Sand at *ψ*_*s,top*_ = −100 cm, **(B)** Loam at *ψ*_*s,top*_ = −659.8 cm, and **(C)** Clay at *ψ*_*s,top*_ = −659.8 cm.

We expanded the benchmark setting to include a sand and clay scenario (Figure 2). Simulation time was increased to 7 days to include times of interest for clay. We used the same initial condition of *ψ*_*s,top*_ = −659.8*cm* for clay. For sand, we opted for a more agronomically relevant *ψ*_*s,top*_ of −100*cm*. For the case of dry sand, Figure 2A, we observe stress onset on day 1 for both modeling approaches. Even the RSS model with a grid of 4.0 cm only reaches a *T*_*cum*_ of 3.9 cm^3^ after 7 days. As the grid is refined, T_*cum*_ is successively reduced to 1.2 cm^3^ for the 0.4 cm grid. For the multi-scale model, we observe an even earlier reduction in transpiration, leading to a *T*_*cum*_ 0.06 cm^3^ at the end of the simulation. Hence, even the 0.4 cm grid overestimates the *T*_*cum*_ by a factor of ≈20 compared to the multi-scale model.

In the dry clay soil, Figure 2C, no stress is simulated while using a 4 cm grid and 44.8 cm^3^ are transpired after 7 days. We see a transpiration reduction starting at 6.4 days for the 3.0 cm grid. Further grid refinement to 0.4 cm decreases the *T*_*cum*_ to 37 cm^3^ after 7 days. The multi-scale model simulates a *T*_*cum*_ of 33.7 cm^3^. Consequently, the difference between the models is smallest for this soil.

## 4. Discussion

Overestimation of RWU under drying conditions using RSS modeling concepts was found to depend on soil discretization for all soils analyzed in the scenarios (Figure 2). Even fine grids (0.2 cm), resulted in 30% overestimation of *T*_*cum*_ compared to the numerical reference solution (Figure 1A). The multi-scale model underestimated the reference solution by 20%. However, we need to keep in mind that the reference is itself a numerical solution. A larger fraction of the total soil volume than in the reference solution is covered by small edge-lengths in the multi-scale model (Supplementary Figure S2). Conceptually, as the multi-scale model assumes equal distribution of root segments within the voxel, it is more likely to still overestimate RWU. Nevertheless, we could demonstrate that the multi-scale model outperforms the RSS approach in accuracy, while being roughly 300 times faster (Figure 1A) and being more stable for different grid sizes (Supplementary Figure S4). Due to the lower computational effort, simulations of large RSA's and multiple RSA's in parallel are still possible on a local computer. We are aware that adaptive grids only refined in areas containing root segments would improve the performance of the RSS model. However, as shown by Schroeder et al. (2009b), use of adaptive refinement with acceptable error margins would improve the speed with an order of magnitude of one at best. It also remains to be investigated whether other numerical methods such as FEM in combination with unstructured grids exhibit similar grid dependencies, and if the continuum assumption is still suited to describe water flow on very fine grids as used in the multi-scale model.

Whether a more detailed representation of *k*_*soil*_ gradients through the demonstrated improvements in rhizosphere soil process descriptions alone leads to more realistic RWU predictions is still debatable. Biophysical processes such as mucilage deposition (Kroener et al., 2014; Carminati et al., 2016; Landl et al., 2021) have been shown to create challenging entanglements in the rhizosphere, which can heavily alter the *k*_*soil*_ gradients. On the other hand, a gap between process descriptions and the current means of measurement methods could be introduced or widened. RSA's derived using MRI or CT can miss a significant proportion of fine roots (Metzner et al., 2015), which could lead to a systematic underestimation of RWU if used in modeling approaches such as the multi-scale model. Missing fine roots would lead to an overestimation of RWU per unit root length for the remaining roots and larger *ψ*_*s*_ gradients around the roots would limit earlier transpiration. As Cowan (1965) shows, such changes in the ratio of total root length to *T*_*pot*_ can significantly alter *ψ*_*s,eq*_, *ψ*_*s,bulk*_, and their daily patterns, making reliable parameterization of this ratio an ongoing challenge. In addition, current measurement methods do not allow soil hydraulic properties to be reliably determined at the rhizosphere scale and we, therefore, lack the possibility to validate simulations.

Despite these challenges, we are convinced that a shift in RWU modeling paradigms for drought conditions and rhizosphere processes, in general, is needed. A new generation of more advanced RWU models is starting to emerge. Most of these approaches utilize simplified local models around the root segments coupled to RSS models defined on a coarse grid. The *ψ*_*RSI*_ is approximated numerically (Mai et al., 2019) or by a local analytical solution based on the Kirchhoff transformation of the 1D radially symmetric Richards equation and a steady-rate (Schroeder et al., 2009a) or steady-state assumption (Koch et al., 2021). In addition, Koch et al. (2021) allow distributing sink terms around root segments over a small radially symmetric tubular support. Beudez et al. (2013) also use a local analytical solution of the linearized form of the Richards equation (Richards, 1931) and additionally apply the superposition principle to account for potential uptake competition due to dense root clusters. Graefe et al. (2019) also extended a cylindrical root model to account for non-regular root distributions. In addition to non-regular root distributions, de Willigen et al. (2018) included partial contact between roots and soil in cylindrical models. Ultimately, it will be these approaches that serve as frameworks to consider rhizosphere processes and upscale them to the RSS. They combine computational efficiency with the option to incorporate rhizosphere-scale information if it becomes available and enable comparisons between simulations and data at this scale.

## Data Availability Statement

The raw data supporting the conclusions of this article will be made available by the authors, without undue reservation.

## Author Contributions

AS, DK, JV, and TS contributed to the conception and design of the study. AS supervised the research activity. DK and TS wrote the manuscript and performed the simulations. Model implementation and development were done by DL and TS. AS, HV, and JV critically reviewed the manuscript. All authors contributed to the article and approved the submitted version.

## Funding

This study was partially funded by the German Research Foundation (DFG) under Germany's Excellence Strategy, EXC-2070 – 390732324 – PhenoRob. TS was funded by the DFG (Grant Number SCHN 1361/3-1). DK was funded by the German Federal Ministry of Education and Research (BMBF) in the framework of the funding initiative Soil as a Sustainable Resource for the Bioeconomy BonaRes, the project BonaRes (Module A): Sustainable Subsoil Management - Soil3; subproject 3 (Grant 031B0515C).

## Conflict of Interest

The authors declare that the research was conducted in the absence of any commercial or financial relationships that could be construed as a potential conflict of interest.

## Publisher's Note

All claims expressed in this article are solely those of the authors and do not necessarily represent those of their affiliated organizations, or those of the publisher, the editors and the reviewers. Any product that may be evaluated in this article, or claim that may be made by its manufacturer, is not guaranteed or endorsed by the publisher.
