# Supplementary material for: Root System Scale Models Significantly Overestimate Root Water Uptake at Drying Soil Conditions
Source: Front Plant Sci. 2022 Feb 14;13:798741. doi: 10.3389/fpls.2022.798741 (PMC8882956; doi:10.3389/fpls.2022.798741)
Supplement: Supplementary file 1 [file Data_Sheet_1.PDF]

# Supplementary Material

## 1 SUPPLEMENTARY TABLES

**Table S1.** Soil hydraulic parameters. $\theta_{res}$ is the residual water content, $\theta_{sat}$ is the saturated water content, $\alpha$, and $n$ are the van genuchten parameters, $K_{sat}$ is the saturated soil hydraulic conductivity and $\lambda$ is the van Genuchten-Mualen parameters.

| Soil type | $\theta_{res}$ | $\theta_{sat}$ | $\alpha$ | $n$ | $K_s$ | $\lambda$ |
|---|---|---|---|---|---|---|
| Sand | 0.045 | 0.43 | 0.15 | 3.0 | 1000 | 0.5 |
| Loam | 0.08 | 0.43 | 0.04 | 1.6 | 50 | 0.5 |
| Clay | 0.1 | 0.40 | 0.01 | 1.1 | 10 | 0.5 |

**Table S2.** Different soil resolutions and their corresponding soil voxel sizes. $VS_x$, $VS_y$, and $VS_z$ refers to the soil voxel sizes in x-, y-, and z- direction respectively, and $VS$ gives an approximated soil voxel size as used in the manuscript for simplicity.

| Soil resolution | Degree of freedom | $VS_x[cm]$ | $VS_y[cm]$ | $VS_z[cm]$ | $VS[cm]$ |
|---|---|---|---|---|---|
| 40 x 40 x 75 | 120000 | 0.20 | 0.20 | 0.20 | 0.2 |
| 26 x 26 x 50 | 33800 | 0.31 | 0.31 | 0.30 | 0.3 |
| 20 x 20 x 38 | 15200 | 0.40 | 0.40 | 0.39 | 0.4 |
| 13 x 13 x 25 | 4225 | 0.62 | 0.62 | 0.60 | 0.6 |
| 10 x 10 x 19 | 1900 | 0.80 | 0.80 | 0.78 | 0.8 |
| 8 x 8 x 15 | 960 | 1.00 | 1.00 | 1.00 | 1.0 |
| 5 x 5 x 10 | 250 | 1.60 | 1.60 | 1.50 | 1.5 |
| 4 x 4 x 8 | 128 | 2.00 | 2.00 | 1.88 | 2.0 |
| 3 x 3 x 5 | 45 | 2.67 | 2.67 | 3.00 | 3.0 |
| 2 x 2 x 4 | 16 | 4.00 | 4.00 | 3.75 | 4.0 |

## 2 SUPPLEMENTARY FIGURES

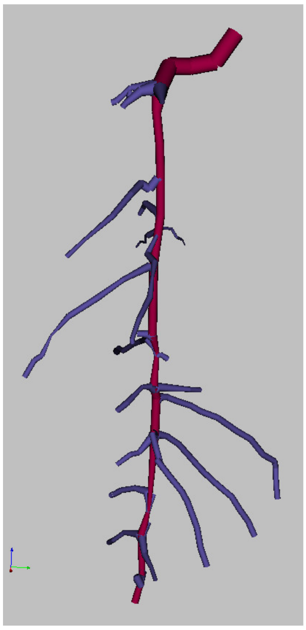

**Figure S1.** Root system architecture of a static 8-day old lupin plant. Red refers to the taproot, purple shows $1^{st}$ order laterals.

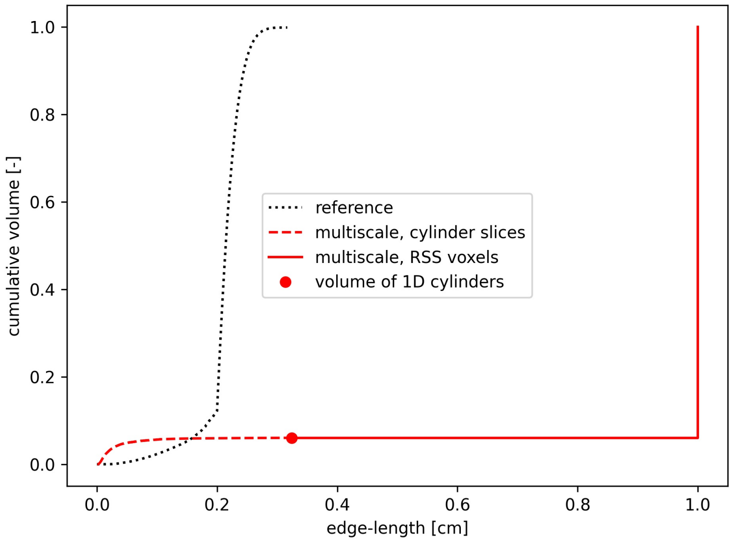


**Figure S2.** Edge-lengths of the grid elements used in the reference solution (black) and the multiscale model (red) against cumulative volume covered by a certain edge-length.

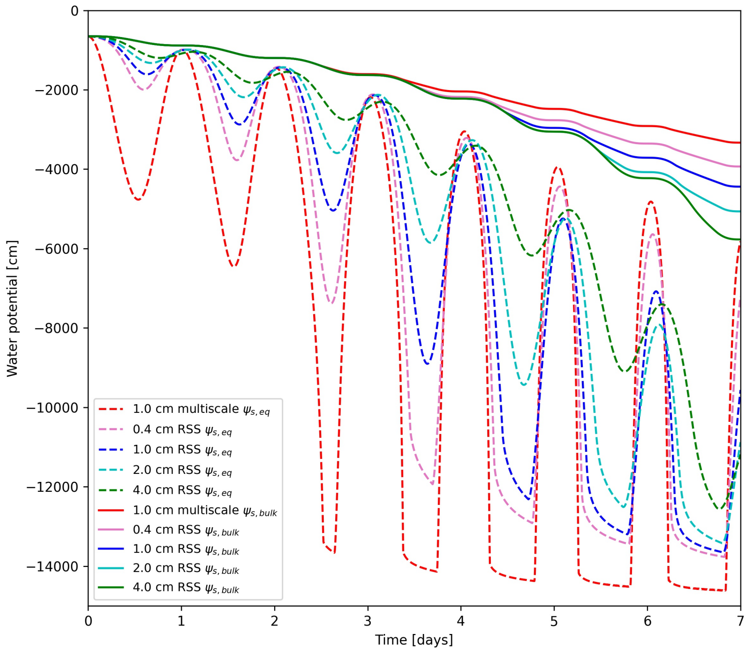


**Figure S3.** Equivalent soil water potential, $\psi_{s,eq}$, (dashed) and mean bulk soil water potential, $\psi_{s,bulk}$, (solid) for multiscale and root system scale (RSS) model at different soil resolutions in the clay scenario at initial $\psi_{s,top} = -659.8$ cm.

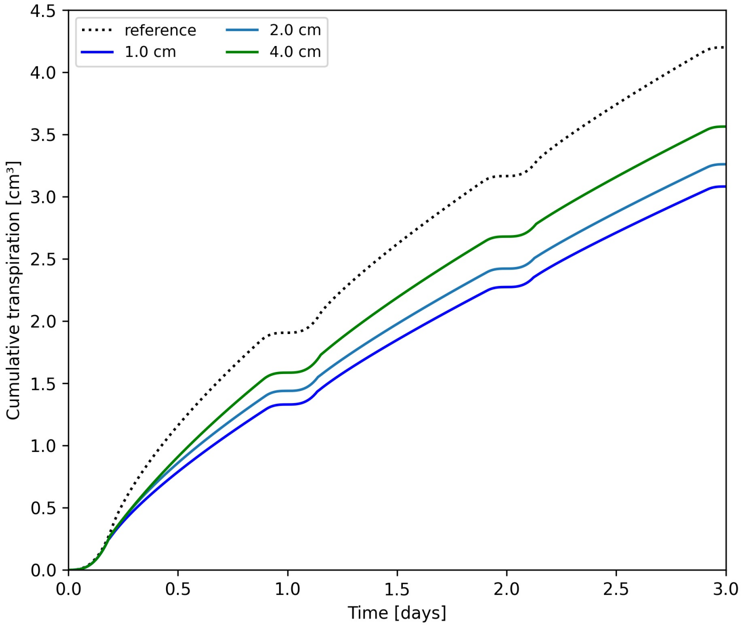


**Figure S4.** Cumulative transpiration of the reference solution (dotted) and the multiscale model at different soil resolutions (solid) for the loamy soil scenario of benchmark C1.2 at initial $\psi_{s,top} = -659.8$ cm.
